# The complete plastid genome of *Lilium regale* E.H.Wilson

**DOI:** 10.1080/23802359.2021.1882913

**Published:** 2021-03-11

**Authors:** Leifeng Xu, Yuwei Cao, Panpan Yang, Jun Ming

**Affiliations:** Institute of Vegetables and Flowers, Chinese Academy of Agricultural Sciences, Beijing, China

**Keywords:** *Lilium regale* E.H.Wilson, plastid, genome sequence

## Abstract

*Lilium regale* E.H.Wilson is a native lily species in western Sichuan of China and an important resource for lily breeding. In this study, the plastid genome of *L. regale* was assembled *de novo* using the next-generation sequencing data. The plastid genome of *L. regale* was 152,998 bp in length, with a typical quadripartite circle structure consisting of a small single-copy region of 17,529 bp, a large single-copy region of 82,375 bp, and a pair of inverted repeats of 26,547 bp each. A total of 137 different genes were predicted, including 84 protein-coding genes, 38 transfer RNA genes, 8 ribosomal RNA genes, and 7 pseudogenes. The overall GC content of the plastid genome was 36.98%. Phylogenetic analysis revealed that *L. regale* is most closely related to *Lilium leucanthum*.

*Lilium regale* E.H.Wilson, belonging to the genus *Lilium* of the family Liliaceae, is widely distributed in western Sichuan of China. It is a precious germplasm for lily breeding due to its high-level resistance to viruses, fungi, and abiotic stresses. Although several nuclear and plastid sequences of *L. regale* were generated to perform phylogenetic studies (Nishikawa et al. 1999; Gao et al. 2013; Wu et al. 2015), the available genetic resource for this species is still limited. In the present study, we generated the complete plastid genome of *L. regale*, and clarified the phylogenetic relationship of *L. regale* with other species in the Liliaceae family.

The sample of *L. regale* was collected from the Mao County, Sichuan province, China (31°35′24′′ N, 103°44′14′′ E). A voucher specimen (CAASL200701) of *L. regale* was deposited at the Institute of Vegetables and Flowers, Chinese Academy of Agricultural Sciences, Beijing, China. Total DNA of *L. regale* was extracted from fresh leaves using the modified CTAB method (Doyle and Doyle 1987). One genomic library was generated according to the manufacturer’s protocol of the TruSeq DNA PCR-Free Library Prep Kit (Illumina, San Diego, CA, USA). The library was sequenced by paired-end sequencing on the Illumina Hiseq X Ten platform (Illumina, San Diego, CA, USA) at Berry Genomics Co., Ltd. (Beijing, China), and 6.38 Gb sequence data was generated. The raw reads were quality trimmed by Trimmomatic (Bolger et al. 2014), and high-quality reads were assembled via NOVOPlasty v2.7.0 (Dierckxsens et al. 2017) with *ribulose-1,5-bisphosphate carboxylase/oxygenase large subunit* (*rbcL*) gene from *L. henryi* (GenBank accession no. NC035570) as the seed. The average coverage depth of the plastid genome was 321×. The plastid genome annotation was performed using CPGAVAS2 (Shi et al. 2019) and GeSeq (Tillich et al. 2017) with default settings followed by manual corrections. The regions with similarity to known protein-coding genes but lacking intact open reading frames were identified as pseudogenes. Simple sequence repeat (SSR) motifs were investigated using the MISA (Beier et al. 2017).

The complete plastid genome of *L. regale* was 152,998 bp in length and consisted of a large single-copy (LSC) region (82,375 bp), a small single-copy (SSC) region (17,529 bp), and two inverted repeat (IR) regions (26,547 bp). The overall GC content of the plastid genome was 36.98%. It encodes 137 genes, comprising 84 protein-coding genes, 38 transfer RNA (tRNA) genes, 8 ribosomal RNA (rRNA) genes, and 7 pseudogenes (*infA*, *rps19*, *ycf1*, *ycf15* (×2), and *ycf68* (×2)). Among these genes, 16 different genes (*trnA-UGC*, *trnI-GAU*, *trnL-UAA*, *trnV-UAC*, *trnK-UUU*, *trnG-UCC*, *petB*, *petD*, *atpF*, *ndhA*, *ndhB*, *rpl2*, *rpl16*, *rps12*, *rps16*, and *rpoC1*) contains one intron, and two genes (*clpP* and *ycf3*) contained two introns. Most of these genes were single-copy genes, whereas 20 genes occurred in double copies, including six protein-coding genes (*ndhB*, *rpl2*, *rps7*, *rps12*, *ycf2*, and *rpl23*), eight tRNAs (*trnA-UGC*, *trnH-GUG*, *trnI-CAU*, *trnI-GAU*, *trnL-CAA*, *trnN-GUU*, *trnR-ACG*, and *trnV-GAC*), and four rRNAs (*rrn4.5*, *rrn5*, *rrn16*, and *rrn23*), and two pseudogenes (*ycf15* and *ycf68*). A total of 105 simple sequence repeats were identified in the complete plastid genome of *L. regale*, of which mononucleotide motifs were the most frequent, and 64.76% were located in non-coding regions.

To confirm the phylogenetic position of *L. regale*, the complete plastid genomes of 19 species in the family Liliaceae were downloaded from the NCBI GenBank database (www.ncbi.nlm.nih.gov/nucleotide/). The sequences were aligned using MAFFT v7.475 (Katoh et al. 2017), and then a maximum-likelihood tree was constructed with 1000 bootstrap replicates using MEGA v7.0 software (Kumar et al. 2016). Phylogenetic analysis showed that *L. regale* exhibited a closest relationship with *Lilium leucanthum* (Figure 1). This study could serve as a valuable genomic resource providing insight into conservation and exploitation and genetic evolution for this ornamental species.

**Figure 1.**
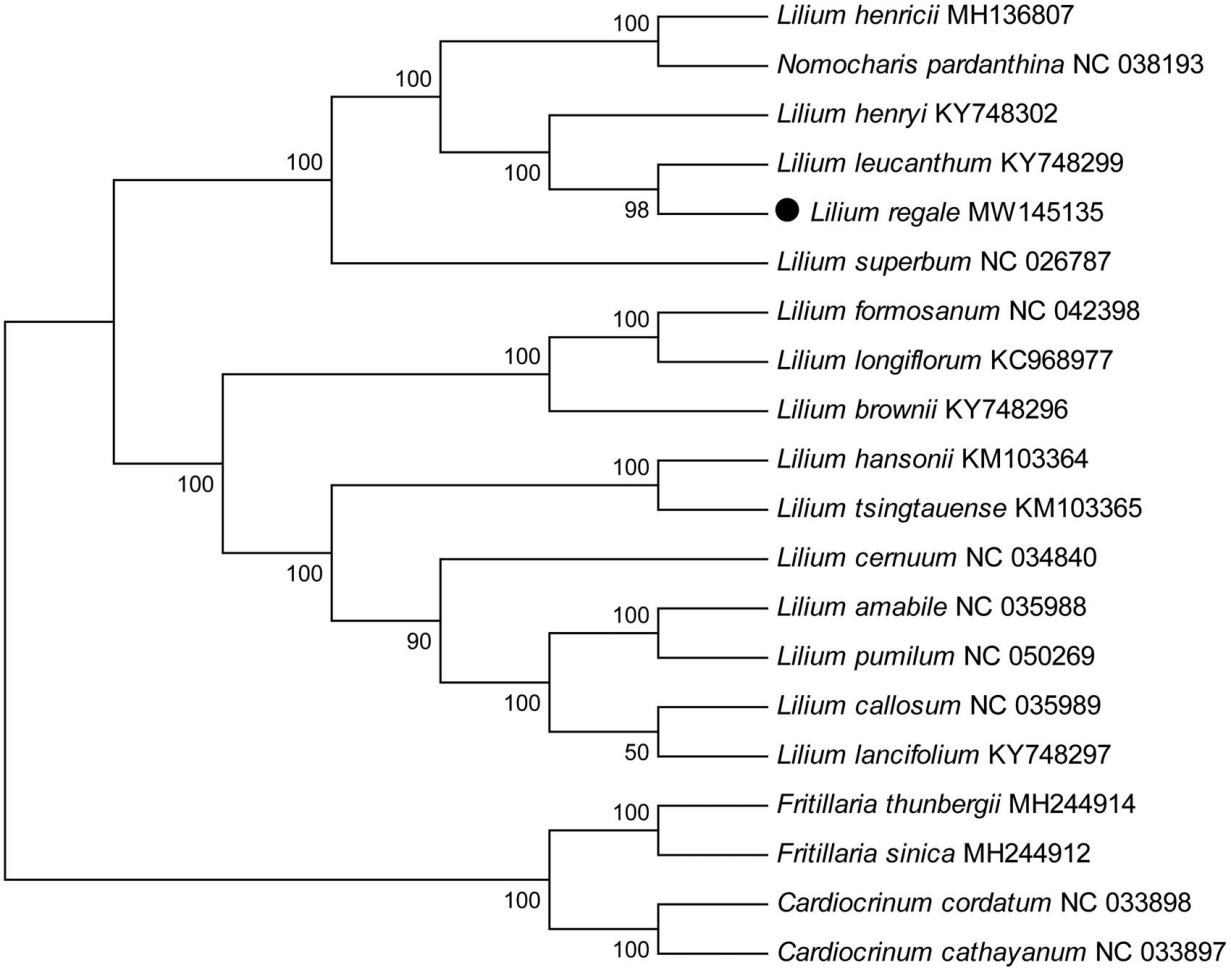
Phylogenetic tree showing the relationship between *L. regale* and 19 species belonging to the family Liliaceae. Numbers on nodes indicate bootstrap values.

## Data Availability

The genome sequence data that support the findings of this study are openly available in GenBank of NCBI at (https://www.ncbi.nlm.nih.gov/) under the accession no. MW145135. The associated BioProject, SRA, and Bio-Sample numbers are PRJNA689397, SRR13347195, and SAMN17207829, respectively.
